# Age is associated with time in therapeutic range for warfarin therapy in patients with atrial fibrillation

**DOI:** 10.18632/oncotarget.10944

**Published:** 2016-07-29

**Authors:** Leiliane Rodrigues Marcatto, Luciana Sacilotto, Francisco Carlos da Costa Darrieux, Denise Tessariol Hachul, Maurício Ibrahim Scanavacca, Jose Eduardo Krieger, Alexandre Costa Pereira, Paulo Caleb Junior Lima Santos

**Affiliations:** ^1^ Laboratory of Genetics and Molecular Cardiology, Heart Institute (InCor), University of São Paulo Medical School, São Paulo, Brazil; ^2^ Clinical Cardiology Division, Heart Institute (InCor), University of São Paulo Medical School, São Paulo, Brazil

**Keywords:** warfarin, TTR, age, atrial fibrillation, polymorphisms, Gerotarget

## Abstract

**Background:**

Warfarin is the most prescribed oral anticoagulant used for preventing stroke in patients with atrial fibrillation. Time in the therapeutic range (TTR) has been accepted as the best method to evaluate the quality of warfarin therapy. The main aim of the present study was to evaluate the impact of variables on the time in the therapeutic range for warfarin therapy in patients with atrial fibrillation from a referral cardiovascular hospital.

**Methods:**

This retrospective study included 443 patients were included (190 patients with age < 65 years and 253 patients with age ≥65 years) from 2011 to 2014 and TTR was computed according to Rosendaal's method.

**Results:**

Patients with age ≥65 years had higher TTR value (67±22%) compared with patients with < 65 years (60±24%) (*p* = 0.004). In a linear regression model, only age ≥65 years emerged as a significant predictor of greater TTR values. In multivariate logistic regression model, the variable age ≥65 years was associated with higher OR for having a TTR higher than the median value (OR = 2.17, *p* < 0.001).

**Conclusion:**

We suggest that the age influenced TTR through greater drug adherence. Strategies for increasing drug adherence might improve quality of warfarin anticoagulation.

## BACKGROUND

Atrial fibrillation (AF) is the most common cardiac arrhythmia and it has a high estimated prevalence of 33.5 million people in worldwide [1, 2]. The prevalence of this disease increases with age from < 0.5% between 40 and 50 years, to 5% to 15% at > 80 years of age [3]. AF may be associated with severe consequences, like stroke with neurological deficit and death [4]. Anticoagulation therapy is the most important measure for preventing stroke in the AF setting.

Warfarin is the most commonly used oral anticoagulant for preventing stroke in patients with AF. However, this drug has a narrow therapeutic index and a large inter-individual variability in dose requirements [5, 6]. Thus, clinicians use the international normalized ratio (INR) for monitoring the warfarin therapy. High values of INR are associated with bleeding while low values of INR are associated with thromboembolism and stroke [7, 8]. INR values are used to calculate the time in the therapeutic range (TTR).

TTR is a measure of quality of anticoagulation therapy and high TTR values mean a good quality of treatment [9, 10]. A study conducted in Europe, included 6,250 patients with AF using warfarin from different countries, showed that TTR levels ranged from 66.0% in Italy to 72.6% in the United Kingdom. In France, Germany, and Italy, less than 50% of the patients had TTR > 70% [11]. Another study, including 377 patients and considering TTR < 60% as a marker of poor quality in the anticoagulation control, showed that 44.3% of the patients had a poor anticoagulation therapy [12].

Age, body mass index, concomitant drugs, patient adherence and genetic factors can influence INR values [13–15]. However, the factors which can significantly influence TTR measures, i.e., warfarin anticoagulation therapy in the long-term are not fully understood [9, 16]. Identification of factors that influence TTR values is important to guide the best clinical approach during the treatment. In this scenario, the main aim of the present study was to evaluate the impact of variables on the TTR for warfarin therapy in patients with AF from a referral cardiovascular hospital.

## RESULTS

Table 1 shows demographic and clinical characteristics of the patients according to age over 65 years. Mean age in the younger group was 56±9 years (*n* = 190) and in the older was 73±6 years (*n* = 253). We observed higher percentages of self-declared White, diabetes mellitus, and time in warfarin therapy in the group of patients with age ≥65 years compared with group of patients with age < 65 years.

**Table 1:** Demographic and clinical characteristics of participating patients according to age over 65 years

Variable	< 65 years *n* = 190	≥ 65 years *n* = 253	*p* value
**Sex, female** (%)	45.8	41.5	0.37
**Self-declared race, White** (%)	59.3	73.9	**0.001**
**Body mass index** (Kg/m^2^)	27.7±5.2	26.9±4.4	0.11
**Smoking** (%)	7.0	4.8	0.33
**Diabetes mellitus** (%)	19.1	28.5	**0.03**
**Hypertension** (%)	88.9	87.4	0.62
**Hyperlipidemia** (%)	41.7	45.5	0.43
**Heart failure** (%)	33.9	29.8	0.36
**Warfarin dose** (mg/week)	28.8±12.5	26.8±11.5	0.08
**Amiodarone use** (%)	18.9	15.9	0.40
**Time in warfarin therapy** (years)	5.0±4.6	6.2±5.0	**0.02**
***CYP2C9* genotypes (IM+PM)** (%)	27.1	27.3	0.97
***VKORC1* genotypes, GG/GA/AA** (%)	47.9/42.6/9.5	43.4/47.8/8.8	0.55

Table 2 shows TTR mean according to age over 65 years. We observed that, in the overall group, patients with age ≥65 years had higher TTR mean than patients with age < 65 years (67±22% and 60±24%, *p* = 0.004, respectively). We also observed higher TTR mean in patients with age ≥65 years than patient with age < 65 years in the female group (66±21% and 61±23%, *p* = 0.04, respectively) and in the male group (68±23% and 59±25%, *p* = 0.01, respectively). The variable self-declared race was not associated with TTR, even in multivariate models. The TTR mean of the White and non-White groups were not different (64±23% and 62±23%, *p* = 0.24, respectively).

**Table 2:** Mean TTR according to age over 65 years

	TTR (%)		
	< 65 years	≥ 65 years	*p* value
**Total group** (*n* = 443)	60 ± 24	67 ± 22	0.004
**Female** (*n* = 192)	61 ± 23	66 ± 21	0.04
**Male** (*n* = 251)	59 ± 25	68 ± 23	0.01

In a multivariate linear regression model, we analyzed the effect of variables, including age, in years, on TTR values (Table 3). We show that age was also associated with TTR values (*p* = 0.02). Supplementary Table 1 shows a linear regression model with the addition of other variables.

**Table 3:** Multivariate linear regression analysis for TTR

Variable	β standardized coefficient	*p* value
**Age**	0.121	**0.02**
**Sex** (male)	0.007	0.88
**Self-declared race** (White)	0.050	0.33
**Body mass index**	0.029	0.57
**Smoking**	−0.018	0.72
**Amiodarone use**	−0.008	0.88
***CYP2C9* genotypes (IM+PM)**	0.006	0.91
***VKORC1* genotypes**	0.006	0.90

In a multivariate logistic regression model, we analyzed TTR median (≥68.0%) as a dependent variable and the following variables as independent: age ≥65 years, sex (male), self-declared race (White), BMI, smoking, amiodarone use, *CYP2C9* predicted metabolic phenotypes (IM+PM) and *VKORC1* genotypes (Table 4). The variable age ≥65 years was significantly associated with having a TTR value above the median (OR = 2.17, 95%CI = 1.43-3.28, *p* < 0.001). Supplementary Table 2 shows a logistic regression model including other independent variables.

**Table 4:** Multivariate logistic regression analysis for over values of TTR median (≥68.0%)

Variables	OR	95% CI	*p* value
**≥ 65 years**	2.17	1.43-3.28	**<0.001**
**Sex** (male)	1.34	0.89-2.01	0.16
**Self-declared race** (White)	1.36	0.87-2.12	0.18
**Body mass index**	1.01	0.97-1.06	0.56
**Amiodarone use**	0.99	0.59-1.67	0.97
***CYP2C9* genotypes (IM+PM)**	1.22	0.78-1.91	0.39
***VKORC1* genotypes**	0.99	0.72-1.37	0.96

## DISCUSSION

In the present study, our main finding was that patients with age ≥65 years had higher TTR value than those with age < 65 years. Interestingly, few studies have showed this same association. A study including 501 Japanese patients to evaluate factors influencing TTR, such as age, gender, antiplatelet drugs, CHADS_2_ score and warfarin dose showed that patients aged ≥70 years had TTR of 77±17% and patients aged < 70 years had TTR of 46±23% (*p* < 0.001) [16]. Another study, including 6,983 patients from diverse regions of the world, showed that patients with age < 73 years had lower TTR mean levels than patients with age ≥73 years (53.6±20.9 *vs* 56.8±21.5, respectively, *p* < 0.001) [9]. Furthermore, Skeppholm et al showed that younger patients spent more time out of therapeutic range than older patients [17].

We suggest that TTR can be influenced by age presumably because older people have higher compliance (drug adherence) than younger. Here, we chose the age according to the definition of elderly person by WHO (World Health Organization) [18]. Indeed, it has been showed that age younger than 65 years was a risk factor for non-adherence to warfarin [19–21]. Witt et al showed that mean age was higher in patients adherent than in patients non-adherent (70.9 years *vs* 63.8 years; *p* < 0.001) [20]. A study examined the medication taking behavior of older adults. They found that age, gender, education level, marital status, living status and health belief affected the medication knowledge and behavior. They also showed that younger patients had a limited knowledge about their disease and this may be a reason for non-adherence [22]. The greatest adherence to treatment by older people might also be influenced for greater severity of illness and successful compliance behavior by caregivers at home [23]. Another study showed that elderly patients were more adherent to medications after hospital discharge, had an increased interaction with the healthcare system (appointments, number of physician interactions), had higher knowledge about the importance of chronic medication management and had a higher level of experience with managing medications [24]. In the present study, we observed difference in time in warfarin therapy, in years (5.0±4.6 *vs* 6.2±5.0), according to age group. However, we showed that this small difference did not affect TTR values in a multivariate analysis.

A recent study showed that TTR is an independent predictor of major adverse cardiovascular events in their cohort of AF patients. Moreover, the authors showed that age were positively associated to major adverse cardiovascular events and a good anticoagulation is associated with a reduction of these events [25].

Sex and self-declared race did not influence the TTR in our analysis. Okumura et al also did not find differences in TTR mean according sex [16]. However, Singer et al showed that male patients had higher TTR than female patients (56.4±21.2 *vs* 53.3±21.3 *p* < 0.001); and Rose et al showed that the group with TTR > 75% had the lowest proportion of females (37.9%) compared to the group with TTR < 60% (46.5%, *p* < 0.001) [9, 26]. Bhandari et al reported that African-American patients spent less time in range compared with White-American patients (32.2% *vs* 42.0%, *p* < 0.001) [27]. Another study showed that African-Americans spent more time in the sub-therapeutic range (INR < 2) [28].

Neither warfarin dose nor *CYP2C9* and *VKORC1* polymorphisms influence TTR in the present study. However, Okumura et al. found that warfarin dose affected TTR, patients with a low dose (< 2.5mg/day) had higher TTR compared to patients with higher dose (≥5.0mg/day) (72±22%; 48±24%; *p* < 0.001). They suggested that age might have affected the dose administered by the physician. Patients aged < 70 years had higher dose (3.5±1.2mg/day) than patients aged ≥70 years (2.7±1.0mg/day). However, after multivariate analysis, they showed that warfarin dose remained an independent predictor of TTR [16]. Importantly, in our study, the variable age did not affect warfarin dose. Some studies showed that *CYP2C9* and *VKORC1* polymorphisms influenced TTR mean during initiation of therapy with warfarin [14, 15, 29]. We did not find this association probably because our patient group were anticoagulated for at least 12 months.

The variables diabetes mellitus, hypertension and heart failure did not influence TTR mean in our study. These variables are components of the CHADS_2_ score, which estimates stroke risk in patients with atrial fibrillation. Other variables compose the score, such as age ≥75 and a history of stroke or transient ischemic attack symptoms [30, 31]. Okuwura et al. found that CHADS_2_ score influenced TTR. Patients with a score ≤ 1 had TTR of 59±27% and patients with a score ≥2 had 68±23%. The authors explained this finding by the fact that the age of the patients with a score ≥2 (73±8 years) was significantly higher than those patients with a score ≤ 1 (65±11 years) (*p* < 0.001) [16]. On the contrary, Singer et al found that patients with higher CHADS_2_ score (≥2) had lower TTR mean levels (score ≤ 2: TTR = 59.3±19.7; score 3-6: TTR < 55.1±21.3; *p* < 0.01) [9]. Furthermore, another study showed that CHADS_2_ score did not influence TTR [26]. In addition, Singer et al. showed that patients with heart failure had lower TTR mean levels (52.9±21.2 *vs* 59.0±20.7 *p* < 0.001) [9] and Rose et al. found that heart failure was associated with poor control (TTR < 60% = 26.1% *vs* TTR > 75% = 20.5% *p* < 0.001) [26].

Our study has some potential limitations. First, we were not able to use SAMe TT2R2 score in patients with kidney and liver diseases, because we excluded these patients. Second, we did not apply questionnaires about drug adherence; thus, we were not able to test our hypothesis that the elderly had more drug adherence. Furthermore, we did not assess diet, which might be different according to the age group. Second, the influence of the variables on TTR depends on the characteristics of the patient sample. Our sample included patients with atrial fibrillation treated for at least 12 months with warfarin in a referral hospital. Our findings can be different from those of others because of the specifics of our casuistic.

White et al, analyzing the outcome of 3,587 patients with warfarin therapy from SPORTIF III and IV, indicated that the risks of death, myocardial function and stroke or systemic embolic event were lower in patients with TTR ≥60% than in those with TTR < 60% [10]. Here, we highlight the importance of studying variables that may influence TTR levels. We showed that age is associated with TTR. We suggest that age influenced TTR through greater drug adherence. Strategies for increasing drug adherence might improve the quality of warfarin anticoagulation.

## MATERIALS AND METHODS

### Patients and study design

This retrospective study included 443 patients with non-valvular AF treated with warfarin for at least 12 months, from 2011 to 2014, from a referral cardiovascular hospital [32]. The Institutional Ethics Committee (Register Number 0804/10) approved the study protocol and written informed consent was obtained from all participants prior to entering the study. The exclusion criteria were patients with chronic liver failure, using other anticoagulant drugs, receiving chemotherapy, and alcoholism. We obtained data regarding current drug use through a standardized interview with a pharmacist and checked electronic medical records.

### Measures and outcomes

We used prothrombin time (PT) for evaluating oral anticoagulant therapy and obtained the INR calculation by the ratio PT of the patient/normal PT controls, elevated to the international sensitivity index [6, 33]. We collected blood samples to assess current INR and DNA analysis. Besides, we checked past INR values in electronic medical records from the 12 months preceding enrollment. We calculated TTR of the past 12 months using the Rosendaal method [34], which uses linear interpolation to assign an INR value to each day between successive observed INR values.

Genotyping of CYP2C9*2 (c.430C > T, rs1799853), CYP2C9*3 (c.1075A > C, rs1057910), and *VKORC1* 3673 (g.1639G > A, rs9923231) polymorphisms was carried out by polymerase chain reaction followed by restriction enzyme digestion [35, 36]. As quality control, 6% of the samples were reanalyzed and gave identical results. Patients were divided into distinct predicted phenotypes: extensive metabolizer (EM: wild-type genotypes for the *CYP2C9* polymorphisms - *1/*1), intermediate metabolizer (IM: heterozygous genotypes for the loss-of-function *CYP2C9* polymorphisms - *1/*2 or *1/*3) and poor metabolizer (PM: mutant homozygous or compound heterozygous genotypes for the loss-of-function CYP2C9 polymorphisms - *2/*2 or *3/*3 or *2/*3) [37].

### Statistical analysis

We present categorical variables as percentages and continuous variables as mean ± standard deviation. We divided patients into groups according to age: patients with < 65 years and patients with ≥65 years [38]. TTR was adjusted for age, sex, BMI (body mass index), and self-declared race. We tested the effect of the age (in years) on TTR values in a multivariate linear regression model including the following independent variables: age, sex, BMI, race, smoking, amiodarone use, *CYP2C9* genotypes (EM, IM or PM), and *VKORC1* genotypes. In addition, we performed a multivariate logistic regression model to evaluate factors associated with values of TTR above median (≥68.0%), including the following variables: age (≥65 years), sex (male), BMI, self-declared race (White), smoking, amiodarone use, *CYP2C9* genotypes (IM+PM), and *VKORC1* genotypes. All statistical analyses were carried out using SPSS software (v. 16.0) and the level of significance set at *p* ≤ 0.05.

## SUPPLEMENTARY MATERIALS


