# Supplementary material for: Age is associated with time in therapeutic range for warfarin therapy in patients with atrial fibrillation
Source: Oncotarget. 2016 Jul 29;7(34):54194–9. doi: 10.18632/oncotarget.10944 (PMC5342334; doi:10.18632/oncotarget.10944)
Supplement: Supplementary file 1 [file oncotarget-07-54194-s001.pdf]

Age is associated with time in therapeutic range for warfarin therapy in patients with atrial fibrillation

Supplementary material

Supplementary Table 1. Multivariate linear regression analysis for TTR

Variable	β standardized coefficient	p value
**Age**	0.120	**0.03**
**Sex** (male)	0.010	0.84
**Self-declared race** (White)	0.043	0.42
**Body mass index**	0.061	0.27
**Smoking**	-0.016	0.75
**Amiodarone use**	-0.001	0.98
***CYP2C9* genotypes (IM+PM)**	-0.012	0.81
***VKORC1* genotypes**	0.034	0.52
**Alcohol**	-0.050	0.34
**Diabetes**	-0.002	0.97
**Hypertension**	-0.024	0.66
**Heart failure**	0.019	0.72
**Hyperlipidemia**	-0.059	0.27
**Time in warfarin therapy** (years)	0.152	**<0.01**

Supplementary Table 2. Multivariate logistic regression analysis for over values of TTR median (≥ 68.0%)

Variables	OR	95% CI	p value
**≥ 65 years**	1.89	1.22-2.93	**0.004**
**Sex** (male)	1.35	0.88-2.07	0.17
**Self-declared race** (White)	1.40	0.88-2.22	0.16
**Amiodarone use**	0.99	0.58-1.71	0.98
***CYP2C9* genotypes (IM+PM)**	1.10	0.69-1.77	0.68
***VKORC1* genotypes**	1.06	0.76-1.48	0.73
**Body mass index**	1.02	0.97-1.07	0.42
**Alcohol**	0.96	0.55-1.67	0.88
**Diabetes**	1.02	0.62-1.70	0.93
**Hypertension**	0.69	0.35-1.37	0.30
**Heart failure**	0.91	0.58-1.43	0.68
**Hyperlipidemia**	1.04	0.67-1.61	0.87
**Time in warfarin therapy** (years)	1.06	1.01-1.11	**0.01**
